# Low Scattering Microstrip Antenna Based on Broadband Artificial Magnetic Conductor Structure

**DOI:** 10.3390/ma13030750

**Published:** 2020-02-06

**Authors:** Muhammad Saleem, Xiao-Lai Li

**Affiliations:** Key Laboratory for Information Science of Electromagnetic Waves (MoE), School of Information Science and Technology, Fudan University, Shanghai 200433, China; 17210720065@fudan.edu.cn

**Keywords:** radar cross-section (RCS), metasurface, microstrip antenna, artificial magnetic conductor (AMC), wideband, metamaterial

## Abstract

In this summary, we have suggested a new technique in which destructive interference principle is incorporated into a chessboard like a reflective screen, and the proposed antenna realizes a remarkable in-band and also out-of-band backscattered energy reduction by using a metasurface (MS). Two different MS unit cells are designed to provide the resonant frequency with a zero-degree reflection phase. Metasurface unit cells are configured in a chessboard-like reflector screen to achieve the reflection phase difference of 180° ± 37° over a broadband range of frequencies to redirect the scattering field into four quadrants. It is implemented to reduce the backscattered energy level of the microstrip antenna, which is based on destructive interference principle. The simulations indicate that the proposed antenna possesses significant backscattered energy reduction from 6 GHz to 16 GHz in both x– and y– polarization and also −10 dB backscattering reduction at antenna working band (7.4–7.8 GHz) is covered. Moreover, the radiation performance is preserved well and artificial magnetic conductor (AMC) unit cells work at different frequencies which are not influenced on the radiation properties. The bistatic performance of the antenna at different frequencies is also presented. Measurements and simulations of the fabricated design coincide well and the proposed design is verified and validated successfully.

## 1. Introduction

Backscattering reduction of the antenna plays a key role in stealth communication design, and in certain stealth platforms, low backscattering strategy indicates where reduced backward electromagnetic energy of the antenna is needed for security. Metamaterials with electromagnetic properties have been widely used in military equipment, wireless communication, medical application, and other related fields [1]. Probably metamaterials constitute the most recent research achievement in the area of new materials and complex media. Artificial materials exhibit surprising and anomalous electromagnetic properties not found in natural materials. A blend of the radiation and scattering sources, the stealth platform antenna is a significant factor in the overall radar cross-section (RCS) that deteriorates the platform stealth performance. In order to decrease the in-band energy spread, an electromagnetic band-gap structure like a mushroom structure is integrated into the antenna. A fractal antenna is suggested to decrease multi-band backscattering energy relative to standard circular patch antenna [2]. The reduction of backscattering can be regulated by adjusting the dielectric thickness and substrate dielectric constant. Two distinct square AMC units, which are independent of polarization, are proposed for the reduction of backscattering based on the concept of destructive interference and cancellation between perfect electrical conductors (PEC) and AMC [3]. A smaller back-scattering high partially reflecting surface antenna plane to decrease back-scattered energy that is surrounded by the metamaterial ground is proposed in [4]. However, this technique increases the backscatter energy, which will deteriorate the antenna radiation performance. Besides, the bandwidth of the aforementioned designs is limited.

By combining a coding metasurface component to design a small scattering antenna, Fabry–Perot could efficiently decrease scattering and maintain high gain, but backscattered energy band is limited [5]. With the preservation of radiation properties, two different AMC components have been intended for low scattering wideband energy [6]. The backscattering of the AMC unit cells is accomplished in-band, but the antenna operates out-of-band. The reduction of broadband backscattering has been accomplished in a flat arrangement containing a mixture of two correctly constructed AMC structures to attain destructive interference between the reflected waves. On the basis of Jerusalem AMC, the 180° difference in phase between the reflected stage curves was acquired [7]. For applications concerning planar antennas, the wide-band artificial magnetic conductor design is characterized by hexagonal unit cells with and without vias with unilayer frequency–selective surface (FSS) [8,9]. The printed bow-tie antenna with ground surface is a new artificial magnetic conductor structure designed for low profile and gain enhancement fractal wide-band. Two types of square ring patch-based unit cells are used to create the ‘0’ and ‘1’ metasurface coding elements to minimize monostatic backscattering [10,11,12].

All the approaches revealed EM material mounting can affect the efficiency of the scattering antennas. On the other side, in-band monostatic backscattered energy reduction is their crucial concern, while out of band RCS may be in the frequency region as well for radar detection. All approaches show that all previously designed models are confined to monostatic RCS reduction models [13,14,15,16,17,18,19,20,21]; there was no discussion of bistatic RCS but in the proposed design bistatic RCS of the antenna is analyzed. A three-layer metamaterial absorber is used for low RCS antenna but the in-band RCS reduction bandwidth is limited. A novel phase gradient metasurface is designed as a superstrate of the antenna to obtain broadband monostatic backscattered energy reduction. A low RCS patch antenna array is designed to analyze the scattering and radiation performance of the antenna without any extra RCS reduction configurations that can be realized by monostatic RCS of the antenna only [22,23,24]. A low scattering antenna is proposed by using holographic techniques that transform the propagating wave into surface waves to reduce the backscattered energy. In this model, a low backscattering circular polarized antenna is proposed using an absorbing surface. A low RCS reflector antenna is designed by replacing metallic components with compact fluorescent lamps [25,26,27]. Using the polarization conversion units, a method based on antenna array is built to reduce the backscattered energy. The polarization reflective metasurface type chessboard is designed to reduce the backscattered energy of the circular patch antenna. To reduce the backscattered energy of the metasurface-based antenna, the elements are orthogonally prescribed in a chessboard configuration with a rotation arrangement of 90°. The antenna phase gradient metasurface, consisting of H-shaped elements, is proposed to reduce backscattering. To control electromagnetic (EM) wave and backscattering, a multi-bit dielectric reflective metasurface is demonstrated [28,29,30,31,32,33].

The main purpose of the design is to reduce the backscattering energy level of the patch antenna and also maintain the radiation performance. We have observed two modes of the antenna. (i) Radiation mode and (ii) scattering mode. In radiation mode, the radiation pattern and backscattered energy of the antenna is analyzed in detail, and in the scattering mode, the backscattered electromagnetic waves based on destructive interference of the reference and the proposed antenna are analyzed. First, we design a reference antenna with metasurface (AMC) then compare the simulated and measured results with the proposed design (after loading the metasurface at the same substrate). After comparing the results, we can observe the radiation performance of the antenna is kept well and in-band/out of band backscattered energy level is reduced dramatically.

In this paper, a new method to reduce the monostatic as well as bistatic RCS of the antenna has been discussed. In the first part, the combination of two metasurface unit cells is used to form a conventional chessboard surface that has four lobes of scattering patterns to be readily realized. The chessboard of the coding AMC impedance surfaces is regarded using metasurface unit cells in an arrangement with AMC configuration; destructive interference is generated as a result of the phase difference of 180° between the reflected fields of each of the two components, and it redirects the scattered fields in four directions. Few AMC unit cells are diffused to integrate the patch antenna to reduce wideband both in-band and out-of-band monostatic and also bistatic backscattered energy while maintaining radiation properties. In our proposed design, we have designed a simple patch antenna to reduce the backscattering (in-band/out-of-band) of the antenna. We have designed the metasurface unit cell (AMC). The reflecting phase difference is around 180° ± 37° between the AMC unit cells in order to achieve a reduction of more than 10 dB from 6.4–11 GHz. Then we configure the AMC unit cells like a chessboard to obtain destructive interference between the reflected wave to reduce the backscattering and the patch antenna is fed via the coaxial probe.

## 2. Theory and Analysis 

The reflecting phase difference of around 180° ± 37° between the building components must be retained during all the works on conventional chessboard design in order to achieve a reduction of more than 10 dB. However, the working principle of the checkerboard is totally based on the interference between the reflected waves generated by PEC and AMC unit cells, in which variance of 180° phases is attained, while the design limits the backscattered energy reduction in the narrow-band. In order to improve the bandwidth, the PEC cells can be substituted by another AMC structure working at a different resonant frequencies. In this analysis, a technique for the low backscattering of antenna has been proposed to overcome the limitation of the conventional chessboards design, and at the same time, it increases the bandwidth of backscatter energy reduction that is discussed in Section I. The design shows the advantages of wider bandwidth monostatic and also bistatic backscattered energy reduction and smaller unit size in this paper. There is a mathematical expression that calculates the backscattered energy of the novel chessboard surface. More than 51% frequency bandwidth of a 180° phase difference is obtained by combining two AMC cells.

(1) $$RCS\;Reduction=10\;\log_{10}\left|\frac{A_{1}e^{j\emptyset_{1}}+A_{2}e^{j\emptyset_{2}}}{2}\right|$$

where $A_{1}$ and $A_{2}$ are the amplitude of AMC1 and AMC2 unit cells with $\emptyset_{1}$ and $\emptyset_{2}$ phase, respectively. The RCS reduction can be calculated by using Equation (1). The 180° ± 37° phase difference retains between 6.2 and 10.7 GHz over the frequency range to achieve a reduction of more than 10 dB in-band and reduction of backscattered energy with regard to the AMC1 and AMC2 phases. It is challenging to reduce backscattered energy at lower frequency because it is necessary to increase the unit cell size, but in the proposed prototype, backscattered energy is also reduced at lower frequencies. 

As mentioned earlier, backscattering reduction can be achieved with a 180° phase difference for the AMC combination. It can be shown that there is a significant reduction in backscattered energy if the phase change maintains an efficient difference of 180°. Meanwhile, the difference in phase is no longer based on resonance. To check the backscattering energy mechanism, we design a comprehensive metasurface array comprised of M × N elements. The overall scattering field is the superposition of the reflected energy from all M × N unit cells when a plane wave usually impinges on such an array. It is assumed that each of the cells of all units has the same pattern of reflection, based on the general principle of the array. The overall reflection can then be defined by Equation (2) [11].

(2) $$E_{rtotal}=P\cdot F_{A}=P\cdot \overset{M-1}{\underset{m=0}{\sum }}\overset{N-1}{\underset{n=0}{\sum }}e^{j\left[km∆x\sin\theta \cos\emptyset +kn∆y\sin\theta \sin\emptyset \left(m,n\right)\right]}$$

where *P* represents the pattern of the elements, array factor is the *F_A_*, and wavenumber is denoted by *k*, *ϕ (m*, *n*) is the phase of (*m*, *n*), and ∆x and ∆y are the space concerning the adjacent components along x– and –y directions, respectively. Elevation and azimuth angles of an incidence are the *θ* and *ϕ*, respectively. Nevertheless, the 180° phase difference cannot be sustained over a wideband as the reflection phase varies with frequency. Typically, a reduction of 10 dB backscattering is set as a requirement for comparison with PEC or antenna of the same length in Equation (3), that is,

(3) $$10log\left({\left|E_{total-reflected}\right|}^{2}/{\left|E_{PEC}\right|}^{2}\right)\leq -10\;dB$$

The phase features are generally used to calculate the backscattered energy reduction bandwidth. Therefore, the broadband behavior of the determined unit cells is predicted to obtain a low scattering property. Therefore, the broadband behavior of the determined unit cells is predicted to obtain a low scattering property. The principle contributions of the article are as follows:

(1) Two different AMC unit cells are selected for chessboard design to accomplish remarkable backscattering reduction compared to prior work.
(2) Novel chessboard is designed for low scattering antenna that is incorporated by diffusing a few AMC unit cells.
(3) Two different metasurface unit cells have been used to create a chessboard for low wideband scattering. So, a notable reduction in backscattering is observed in the designs.
(4) The physical interpretation is given to justify such broadband design.
(5) Key steps are introduced to synthesize the broadband backscattering reduction surface for the proposed design procedure.
(6) In-band and out-of-band backscatter energy reduction of the proposed antenna is accomplished.

## 3. Chessboard Configuration and Analysis

**Figure 1:**
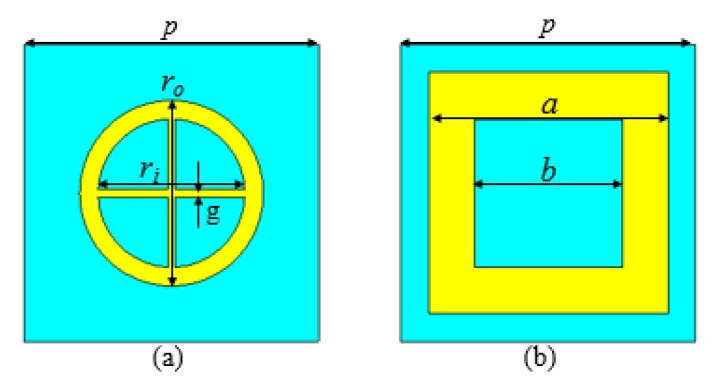
Structure of metasurface unit cells. (**a**) artificial magnetic conductor (AMC1) unit cell. (**b**) AMC2 unit cell.

The proposed AMC unit cells consist of two metal layers separated by a substrate with a thickness of 2.4 mm, a loss tangent of 0.025, and a dielectric constant of 4.3. Figure 1 depicts the structure of two AMC unit cells consisting of a rectangular and ring patch, the bottom one being a metallic ground that is printed on an FR-4 substrate.

**Figure 2:**
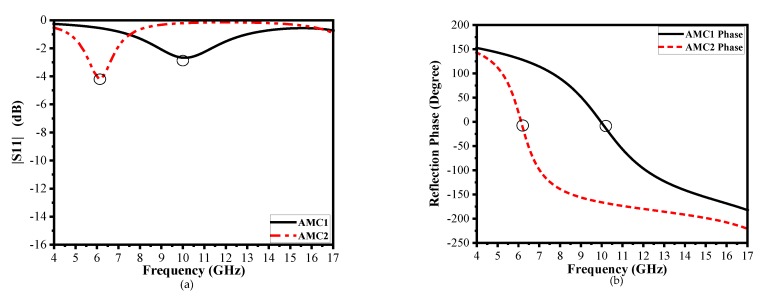
(**a**) Simulated |S11| of two AMC unit cells. (**b**) Simulated results of reflection phase vs. frequency of two AMC unit cells.

The metasurface unit cell design parameters are *p* = 8 mm, *r_o_* = 5 mm, *r_i_* = 3.99 mm, *g* = 0.20 mm, *a* = 6.5 mm, and *b* = 4 mm. Figure 2a illustrates the |S_11_| of both AMC unit cells, which have different resonant frequencies to obtain a wideband effective phase difference. The resonant frequencies of AMC1 and AMC2 are 10.1 GHz and 6.13 GHz, respectively. However, the reflection magnitude is higher because of the use of lossy-substrate (FR-4) to obtain a lower frequency resonant of the AMCs unit cells with an effective phase difference (so that there is little absorption of the energy); is shown in Figure 2a. The patch size primarily governs the reflective phase of these fundamental unit cells in a certain frequency band. Analysis and optimization of the parameters to achieve the reflection phase difference around 180° between two units cells in the broadband frequency range have been carried out by means of CST Microwave Studio software. Unit cell boundaries and floquet ports are used in CST Microwave Studio to create infinite periodic array simulations.

Figure 2b illustrates the reflection phase of two AMC unit cells in a broadband frequency range from 4 GHz to 15 GHz, and demonstrates an efficient phase difference of 180° ± 37° to obtain a wideband reduction of more than −10 dB backscattered energy reduction from 6.4 to 11 GHz.

(4) $$\left|e^{j\emptyset_{1}}+e^{j\emptyset_{2}}\right|=\;\sqrt{2\left(1+\cos\left(\emptyset_{1}-\emptyset_{2}\right)\right)}\leq 0.6325$$

**Figure 3:**
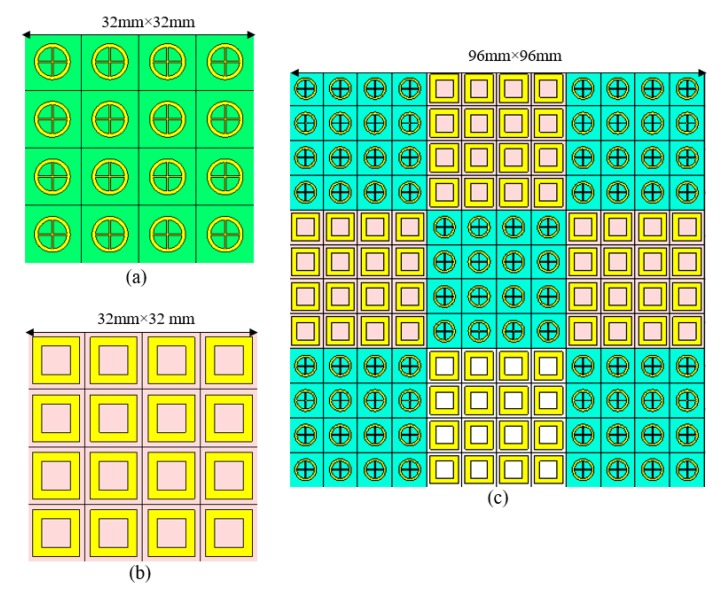
AMC unit cell arrangements in two different chessboards. (**a**) 4 × 4 tiles are arranged with AMC1 unit cell, and the block array size is 32 mm × 32 mm. (**b**) 4 × 4 tiles are arranged with AMC2 unit cell, and the block array size is 32 mm × 32 mm. (**c**) Two AMC unit cells are arranged like a chessboard, an array block size of 96 mm × 96 mm.

Reflection phase difference [3] is calculated by applying Equation (4). A reduction of more than 10 dB RCS can be achieved when the phase difference between two AMC unit cells spans (180° ± 37°) from 143° to 217°, as demonstrated in Figure 2b. Figure 3a,b illustrate the layout of the AMC tiles, which contains 4 × 4 identical unit cells because of interference between reflected waves generated by metasurface unit cells, and phase difference of 180° ± 37° is achieved. The proposed metasurface can then be constituted of a 3 × 3 array block in a chessboard configuration to redirect the scattering energy toward four quadrants in the wide frequency band, as shown in Figure 3c.

## 4. Design of Proposed Low Scattering Antenna

**Figure 4:**
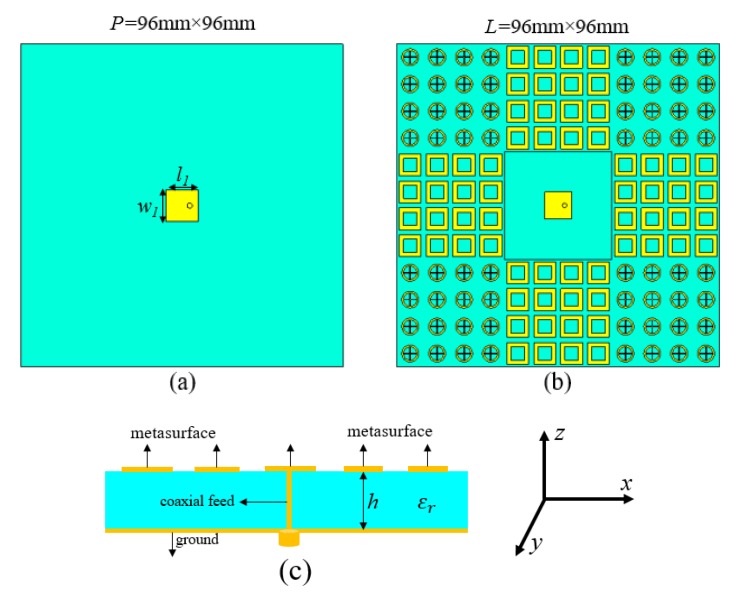
Structure and configuration of reference and proposed antennas. (**a**) The front outlook of the reference antenna. (**b**) The front outlook of the proposed metasurface chessboard with the antenna. (**c**) General configuration of the proposed prototype.

The proposed configuration for the patch antenna is revealed in Figure 4. The proposed pattern consists of a 3 × 3 array block that is mounted around the patch antenna. The microstrip antenna and AMC unit cells are printed on the FR-4 slab, with dielectric constant and loss tangent 4.3 and 0.025, respectively. The new antenna has a patch size of w1 = 8 mm, l1 = 8 mm, and a total dimension of 96 mm × 96 mm. Besides, a conventional antenna is chosen as the reference antenna with PEC, as shown in Figure 4a.

**Figure 5:**
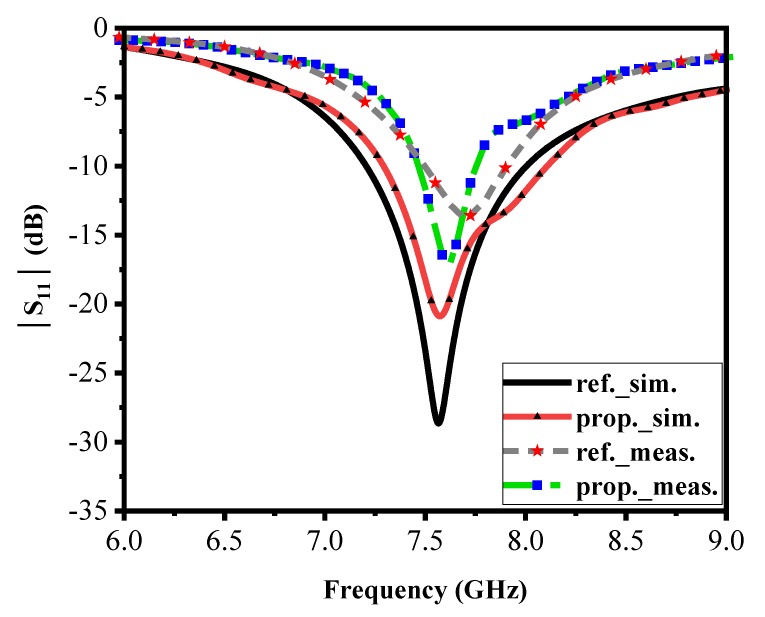
The measurements and simulations |S_11_| of the reference and the proposed antenna.

Due to the small influence of AMC patches, the resonance frequency of the proposed antenna is shifted to a lower frequency. Furthermore, the simulated and measured |S11| is given for the reference and proposed antenna at 7.6 GHz and 7.57 GHz, respectively—this is shown in Figure 5.

**Figure 6:**
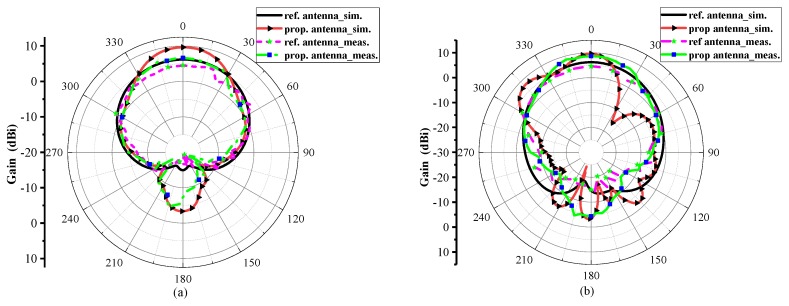
Measured and simulated results of radiation pattern of reference and proposed antenna at 7.6 GHz. (**a**) Measured and simulated radiation patterns in the xoz plane. (**b**) Simulated and measured results of the radiations pattern in the yoz plane.

**Figure 7:**
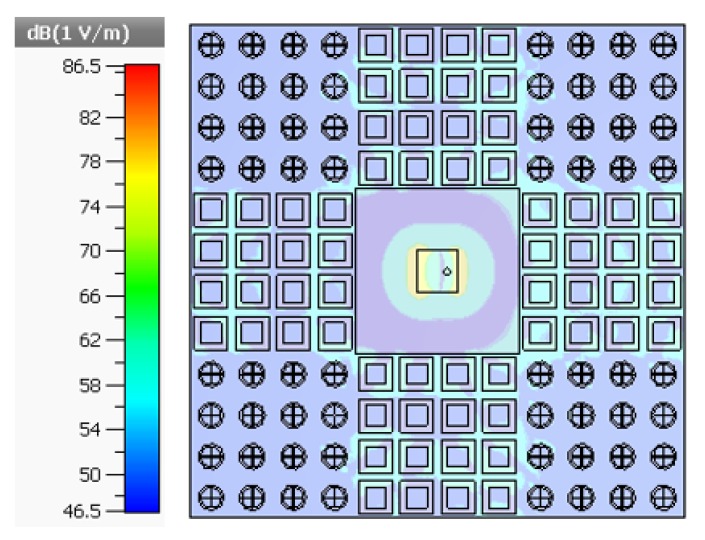
E-field distribution of maximum in-band radar cross-section (RCS) reduction at 7.6 GHz.

**Figure 8:**
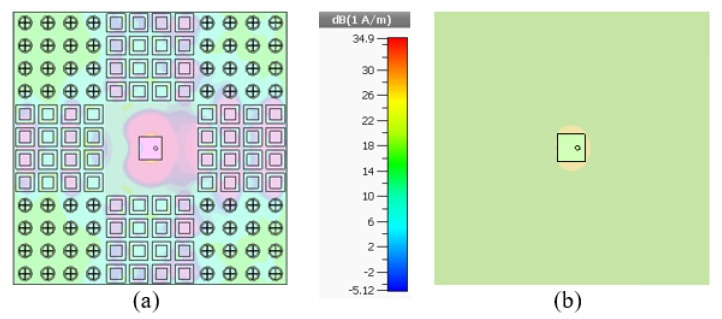
Current distribution on proposed and reference antenna at 7.6 GHz. (**a**) proposed antenna. (**b**) reference antenna.

Figure 6a, b display the simulated and measured results of the radiation performance of reference and proposed antennas at their resonant frequencies in both H–plane and E–plane, respectively. The main lobe direction of the proposed antenna is normal (0 degrees), and the optimum gain is 7.38 dB, that is 1.19 dB above the reference antenna and the radiation directivity coincide well with each other in normal direction (0 degrees). The optimum gain in both polarizations is also maintained. Measured results reveal the excellent agreement between the simulation and measurements of the reference and the proposed antenna in the anechoic chamber. Figure 7 illustrates the distribution of electric fields in which both AMC arrays have a different distribution of electric fields owing to their anti-phase reflection characteristics at 7.6 GHz and also maximum in-band backscattering energy reduction is realized at this frequency. It is verified that reflective wave in-phase and out-of-phase are working properly. The current distribution of the reference and proposed antennas with/without AMC loading is shown in Figure 8.

**Figure 9:**
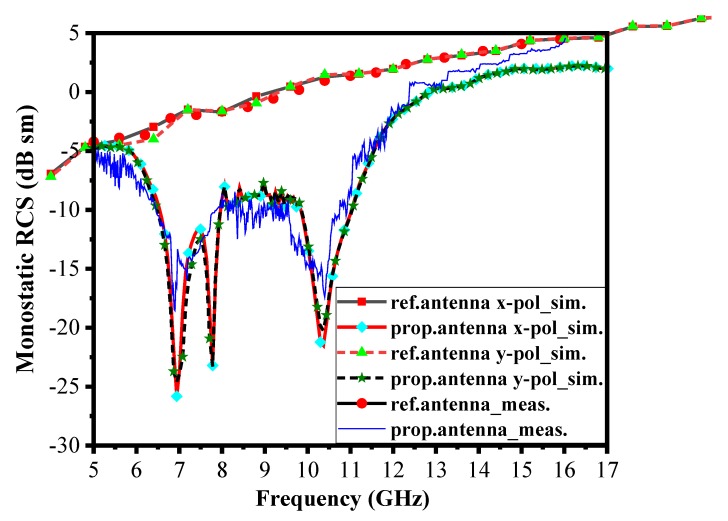
Simulations and measurements of the monostatic backscattering of the reference and proposed antenna.

The monostatic RCS antenna with coding AMC layer as a chessboard reflector for normal incidence was measured. Two traditional horn antennas were used to cover the operating frequency band from 6 GHz to 16 GHz. One of the horn antennae operated as a transmitter, and the other was responsible for the reception, acting as a receiver. The first reference antenna was mounted on a rotary platform and results were measured. After that, the proposed antenna with coding AMC was replaced with a reference antenna and results were investigated. The monostatic backscattering of a reference antenna was reduced by almost −10 dB in both x– and y– polarization, from 6.4 GHz to 11 GHz, and the overall monostatic backscattered energy reduction occurred from 6 to 16 GHz, as shown in Figure 9.

**Figure 10:**
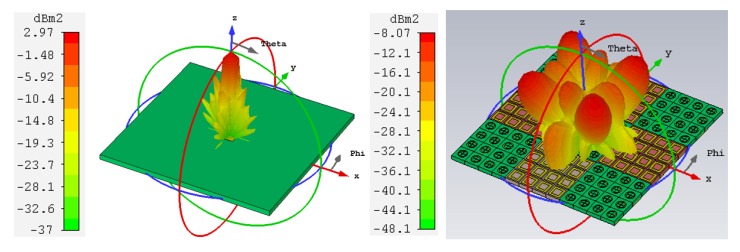
3-D monostatic backscattered energy of the reference and proposed antennas at 10.4 GHz.

Figure 10 illustrates the 3-D monostatic RCS reduction at 10.4 GHz. Bistatic backscattering of both the reference and the proposed antennas were simulated at two different frequencies, and results are compared in both polarizations.

**Figure 11:**
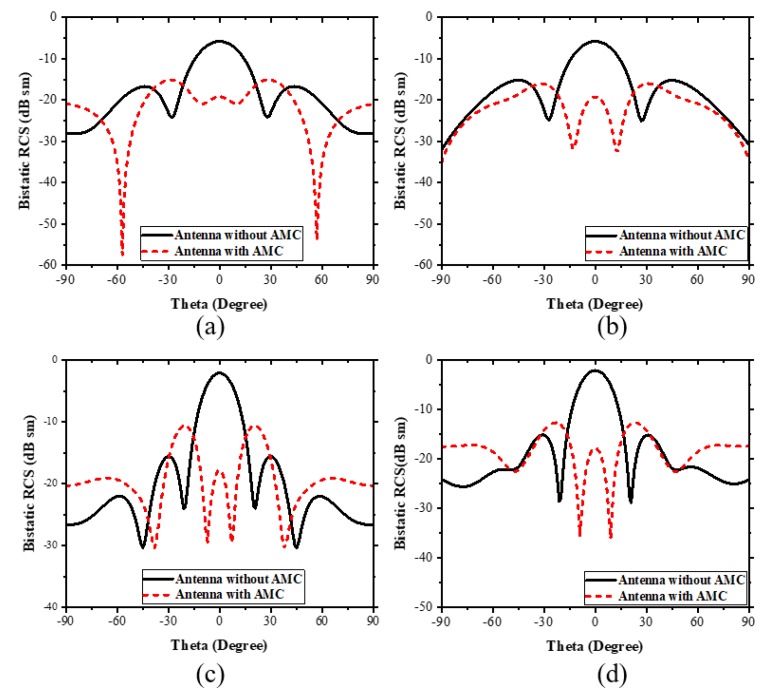
2-D Bistatic backscattering pattern at 7.6 GHz and 10.4 GHz. (**a**) Pattern 2-D RCS in the xoz plane at 7.6 GHz. (**b**) Pattern 2-D RCS in the yoz plane at 7.6 GHz. (**c**) Pattern 2-D RCS in an xoz plane at 10.4 GHz. (**d**) Pattern 2-D RCS in the yoz plane at 10.4 GHz.

The bistatic RCS was efficiently reduced due to the principle of phase cancellation of coding AMC. In Figure 11a bistatic backscattered energy of the proposed prototype was reduced in the angular plane, the backscattering in the xoz plane −69° ≤ θ ≤ ±38°, −21° ≤ θ ≤ +21°, and (b) in the yoz plane, −90° ≤ θ ≤ −37°, −22° ≤ θ ≤ +22° and +37° ≤ θ ≤ +90°. Furthermore, (c) in the xoz plane, −15° ≤ θ ≤ +15° and (d) in the yoz plane, −16.4° ≤ θ ≤16.6°. Bistatic backscattered energy was considerably reduced between these angles.

**Figure 12:**
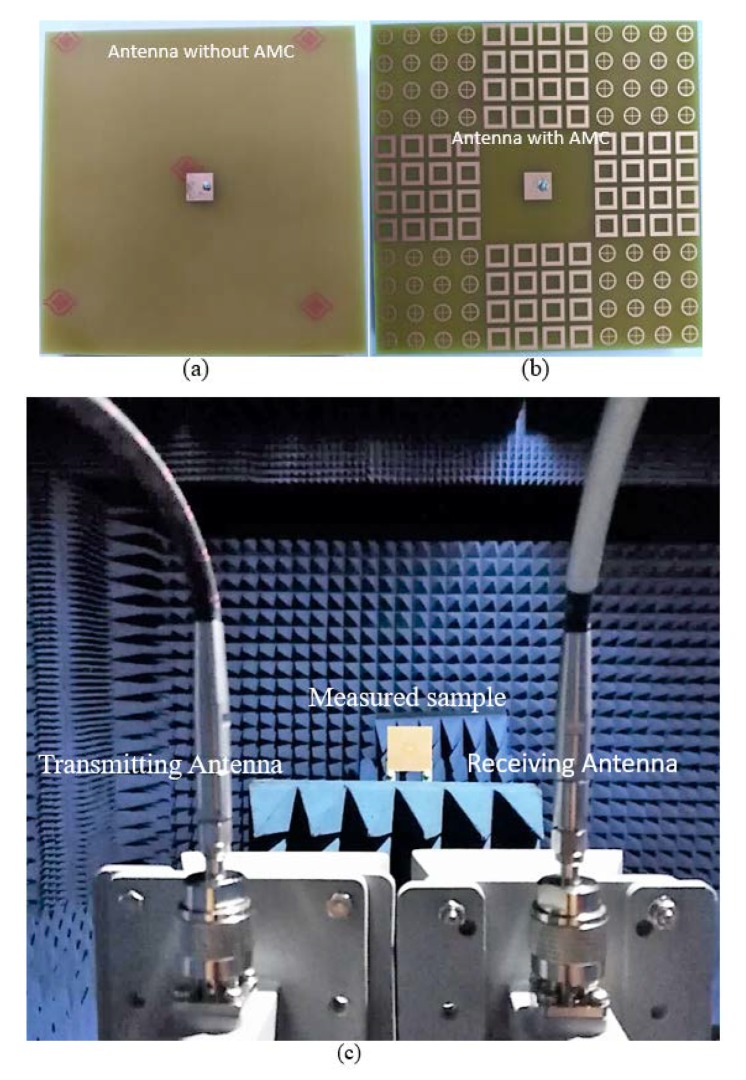
Prototypes of proposed antenna. (**a**) Fabricated reference antenna. (**b**) Fabricated proposed prototype. (**c**) The proposed prototype in a microwave chamber for measurements.

Figure 12 shows the pictures of the fabricated antennas fed by a coaxial probe. The vector network analyzer Agilent N5227A was used to obtain the experimental results of the reference and proposed antennas. The distance between the porotypes and antennas to calculate the far-field was adjusted according to, $r=2D^{2}/\lambda$ where *D* is the dimension of the radiating object. The measured resonant frequency of the reference and proposed antennas were 7.69 GHz and 7.62 GHz, respectively. Both resonant frequencies shifted to a higher frequency. This minor error was caused by the tolerance of measurement and fabrication. Only monostatic backscattered energy of both reference and the proposed antennas were evaluated due to experimental limitations.

## 5. Conclusions

In this summary, we proposed a novel approaches to design low scattering antenna for broadening the bandwidth of monostatic and bistatic backscattered energy diminution. Two different coding AMC unit cells were designed, and the proposed antenna have been integrated into the AMC chessboard substrate by diffusing few AMC patches and destructive phase differences within 6.4–11 GHz to get more than 10 dB backscattering reduction. Maximum backscattered energy reduction reached more than 20 dB at 7.2 GHz 10.4 GHz for both polarizations, respectively. However, the overall backscattered field is reduced from 6 GHz to 16 GHz. Metasurface unit cells and antenna operating frequency is different, but radiation performance is preserved successfully. The proposed design could be a good candidate for monostatic and bistatic backscattered field reduction.
